# Blocking Thromboxane-Prostanoid Receptor Signaling Attenuates Lipopolysaccharide- and Stearic Acid-Induced Inflammatory Response in Human PBMCs

**DOI:** 10.3390/cells13161320

**Published:** 2024-08-08

**Authors:** Vinothkumar Rajamanickam, Cyrus V. Desouza, Romilia T. Castillo, Viswanathan Saraswathi

**Affiliations:** 1 Division of Diabetes, Endocrinology and Metabolism, Department of Internal Medicine, University of Nebraska Medical Center, Omaha, NE 68198, USA; vrajamanickam@unmc.edu (V.R.); cdesouza@unmc.edu (C.V.D.); rocastillo@unmc.edu (R.T.C.); 2 Research Service, VA Nebraska-Western Iowa Health Care System, Omaha, NE 68105, USA

**Keywords:** thromboxane A2, inflammation, lipopolysaccharide, stearic acid, PBMC, ROCK

## Abstract

Inflammation is implicated in the etiology of obesity-related diseases. Thromboxane-prostanoid receptor (TPR) is known to play a role in mediating an inflammatory response in a variety of cells. Gut-derived lipopolysaccharide (LPS), a TLR4 agonist, is elevated in obesity. Moreover, free fatty acids (FFAs) are important mediators of obesity-related inflammation. However, the role and mechanisms by which TPR regulates the inflammatory response in human immune cells remain unclear. We sought to determine the link between TPR and obesity and the role/mechanisms by which TPR alters LPS- or stearic acid (SA)-induced inflammatory responses in PBMCs. Cells were pre-treated with agents blocking TPR signaling, followed by treatment with LPS or stearic acid (SA). Our findings showed that TPR mRNA levels are higher in PBMCs from individuals with obesity. Blockade of TPR as well as ROCK, which acts downstream of TPR, attenuated LPS- and/or SA-induced pro-inflammatory responses. On the other hand, TPR activation using its agonist enhanced the pro-inflammatory effects of LPS and/or SA. Of note, the TPR agonist by itself elicits an inflammatory response, which was attenuated by blocking TPR or ROCK. Our data suggest that TPR plays a key role in promoting an inflammatory response in human PBMCs, and this effect is mediated via TLR4 and/or ROCK signaling.

## 1. Introduction

Worldwide, 537 million adults have been diagnosed with diabetes. This alarming number is expected to rise to 700 million by 2045 [1]. The obesity epidemic has increased the prevalence of diabetes, making diabetes one of the leading causes of death in the U.S. The excess adipose tissue (AT) seen in obesity disturbs cellular homeostasis and upregulates the production of inflammatory markers, including tumor necrosis factor α (TNF-α), monocyte chemoattractant protein-1 (MCP-1), and interleukin 6 (IL-6). Along with the increased production of cytokines, AT promotes the production of hormones and non-esterified fatty acids [2]. These mediators facilitate oxidative stress, a pro-inflammatory state impairing beta cell function, and insulin resistance [3]. Targeting the initiation steps of the inflammation cascade presents a therapeutic target for obesity-induced insulin resistance and hyperglycemia. 

Thromboxane A2 (TXA2) is a lipid mediator in the arachidonic acid signaling system [4]. In an inflammatory state, the enzymes phospholipase A2 and cyclooxygenase (COX-1/COX-2) facilitate the cleavage of arachidonic acid and the conversion to prostaglandin H2 (PGH2). PGH2 is further metabolized into TXA2 by the enzyme thromboxane A synthase (THAS) [4]. TXA2 exerts its biological activity via the transmembrane G-protein-coupled receptor, the thromboxane-prostanoid receptor (TPR), also known as the TXA2 receptor. Although platelet activation and vasoconstriction are two major physiological functions of TPR, it has received significant attention in the past two decades for its pathophysiological role in many diseases. It has been extensively studied for its role in altering the inflammatory processes in endothelial cells [5,6,7]. However, little is known regarding the role of TPR in mediating the inflammatory response in immune cells. Of note, immune cells like macrophages are a significant source of TXA2 [8,9] and express TPR [10]. A study by Hartney et al. has shown that TPR signaling is required for fibronectin-induced matrix metalloproteinase 9 production by macrophages [11]. TPR antagonism inhibited the LPS-induced inflammatory response in rat peritoneal macrophages [12]. Moreover, TPR exerts a pro-inflammatory effect in microglial cells [13,14]. However, the link between immune cell TPR and obesity remains unknown. Moreover, the role and mechanisms by which immune cell TPR exerts an inflammatory response upon treatment with obesity-related factors, in particular LPS and FFA, remain unclear. 

Human peripheral blood mononuclear cells (PBMCs) comprise monocytes, macrophages, and lymphocytes. PBMCs represent a clinically relevant model system to study the role of the immune system in the development of many diseases. LPS is an endotoxin found on the membrane of gram-negative bacteria. The circulating levels of gut-derived LPS are increased in obesity [15]. Moreover, obesity is characterized by an increase in AT lipolysis and a release of FFA from the AT. The LPS-induced inflammatory response is initiated by the activation of toll-like receptor 4 (TLR4), which induces the release of inflammatory cytokines from PBMCs [16]. Similarly, saturated fatty acids are reported to promote an inflammatory response via TLR4 [17]. However, the role and mechanisms by which TPR mediates the pro-inflammatory effects of these obesity-related factors, in particular LPS and FFAs, in human immune cells are still unclear. 

The objectives of this study are to assess the link between PBMC TPR and human obesity and determine the role and mechanisms by which TPR mediates the pro-inflammatory effects in human immune cells, in particular PBMCs. 

## 2. Materials and Methods

### 2.1. Chemicals 

SQ29548 (SQ) (#19025), a TPR antagonist, Ozagrel (OZA) (#70515), a selective inhibitor of THAS, and BM567 (BM) (#10155), a dual-acting agent that inhibits both TPR and THAS, were used to block TXA2 signaling and were obtained from Cayman Chemical (Ann Arbor, MI, USA). [1S-[1α,2α(Z),3β(1E,3S*),4α]]-7-[3-[3-hydroxy-4-(4-iodophenoxy)-1-butenyl]-7-oxabicyclo [2.2.1] hept-2-yl]-5-heptenoic acid (IBOP) (#19600), a TXR agonist, was also obtained from Cayman Chemical (Ann Arbor, MI, USA). Y-27632 2HCl, a selective ROCK1 and ROCK2 inhibitor (#1293823), was obtained from Biogems (Westlake Village, CA, USA). The thromboxane B2 ELISA kit (#501020) and the LDH Cytotoxicity Assay kit (#601170) were obtained from Cayman Chemical (Ann Arbor, MI, USA). Lipopolysaccharide (#L2630) and stearic acid (#N-18A-AU27-T) were purchased from Sigma-Aldrich (St. Louis, MO, USA) and Nuchek Prep (Elysian, MN, USA), respectively.

### 2.2. Human Subjects

Study 1. Study Design and Patient Population. Blood samples were collected from normal subjects (BMI < 30) and individuals with obesity (BMI > 30). Peripheral blood mononuclear cells (PBMCs) were collected to detect the mRNA levels of TPR. This study was approved by the institutional review board of the VA Nebraska-Western Iowa Health Care System. Informed consent was obtained from all subjects involved in the study. The characteristics of the study subjects are shown in Table 1.

Inclusion and exclusion criteria were applied while choosing the subjects for the study. The inclusion criteria were as follows: (1) age 19 to 75; (2) lean/overweight group: BMI between 20 and 29.9 kg/m^2^; obesity group: BMI 30–55 kg/m^2^; (3) subjects should be on a stable dose of any medications for at least two months. The exclusion criteria were as follows: (1) patients currently taking NSAIDs more than 3/week on a prescription basis or taking a daily dose of NSAID; (2) history of diabetes and patients taking diabetes medications, as these drugs may alter AT metabolic functions; (3) history of uncontrolled hypertension defined as >160 systolic and 95 diastolic on medication; (4) history of renal disease with GFR < 60; (5) history of hepatic failure or AST/ALT > three times the normal range; (6) patients with active cancer within the last 2 years except for skin cancers; (7) patients with acute illness needing hospitalization within the last 2 months; (8) patients with acute inflammation; (9) patients with cardiovascular events such as myocardial infarction, stroke, amputation, or unstable angina within the last six months; (10) pregnancy; and (11) presence of psychosis, suicidal ideations, untreated major depression, dementia, or history of stimulant dependence/substance abuse. The study was approved by the institutional review board at the VA Nebraska-Western Iowa Health Care System (NWIHCS). All participants provided informed consent.

Study 2. Blood samples collected from this study were used to isolate PBMCs to perform ex vivo mechanistic studies. This study was approved by the institutional review board of the VA Nebraska-Western Iowa Health Care System and the University of Nebraska Medical Center. Informed consent was obtained from all subjects involved in the study. 

### 2.3. PBMC Isolation and Culture 

Heparinized peripheral blood was collected from all study subjects. PBMCs were isolated from whole blood by Ficoll-Paque Plus (#17144002, Cytiva, MA, USA) density gradient centrifugation at 400× *g* for 35 min at 20 °C. Isolated cells were treated with RBC lysis buffer (#00-4333-57, Invitrogen, CA, USA), washed with PBS, and then resuspended at 1 × 10^6^ cells/mL in RPMI 1640 (Cytiva, MA, USA) medium containing 5% fetal bovine serum (R&D system, Minneapolis, MN, USA). After an overnight incubation, PBMCs were treated with various agents. Media supernatants were collected at 6 h and 24 h for further analyses. 

### 2.4. Real-Time Quantitative Polymerase Chain Reaction

For gene expression analysis, total RNA was isolated from human PBMCs using Trizol (#15596018, Thermo Fisher Scientific, Waltham, MA USA), and cDNA was synthesized using 5X iScript reverse transcription supermix (#1708841, Bio-Rad, Hercules, CA, USA). We conducted real-time polymerase chain reaction (PCR) using iQ Supermix (#1708860, Bio-Rad, Hercules, CA, USA) to identify the mRNA level of *TXA2R* (encoding for TPR). To quantify gene expression, we used the ΔΔCT method and normalized the values to 18s ribosomal RNA. Human *TXA2R* (#Hs00169054_m1) and 18S (#Hs99999901_s1) primers were obtained from Thermo Fisher Scientific (Waltham, MA, USA).

### 2.5. Enzyme-Linked Immunosorbent Assay (ELISA) 

Cell culture supernatants collected at 6 h and 24 h were centrifuged at 1200 rpm at 4 °C for 3 min to remove cell debris. The protein levels of tumor necrosis factor-alpha (TNFα, #555212), interleukin-1β (IL-1β, #55753), monocyte chemoattractant protein-1 (MCP-1, #555179) (BD bioscience, San Jose, CA, USA) and macrophage inflammatory protein-1 alpha (MIP-1α, #DY270, R&D system, Minneapolis, MN, USA) were analyzed using ELISA kits. Briefly, microplates were coated with a diluted capture antibody and incubated at 4 °C overnight. Then, plates were washed 3 times using a wash buffer. Plates were blocked by adding 200 µL of assay diluent for 1 h. Then diluted samples and standards were added, sealed, and incubated for 2 h at room temperature. After 3 washes, plates were incubated with detection antibodies for 1 h and then enzyme SAv-HRP for 1 h. After washing, plates were incubated with a TMB substrate solution for 30 min. After color development, 50 µL of stop solution was added to each well, and absorbance was measured at 450 nm according to the manufacturer’s instructions using the Molecular Devices SpectraMax M5 Microplate Readers (San Jose, CA, USA).

### 2.6. Thromboxane B2 Assay

The levels of thromboxane B2 were determined in the media collected from PBMCs using the thromboxane B2 ELISA kit (Cayman Chemical, Ann Arbor, MI, USA).

### 2.7. LDH Cytotoxicity Assay 

The levels of LDH activity in the cell culture supernatants were determined using the LDH cytotoxicity assay kit (Cayman Chemical, Ann Arbor, MI, USA).

### 2.8. Statistical Analysis 

All of the quantitative variables are presented as the mean ± SEM. Differences among various groups were analyzed using the Student’s *t*-test to compare two groups. A one-way analysis of variance followed by Tukey’s post-hoc analysis was used for multiple comparisons. Statistical analysis was performed by GraphPad Prism 10 software. A *p*-value < 0.05 was considered significant.

## 3. Results

### 3.1. TPR Expression Is Increased in PBMCs in Individuals with Obesity

**Figure 1:**
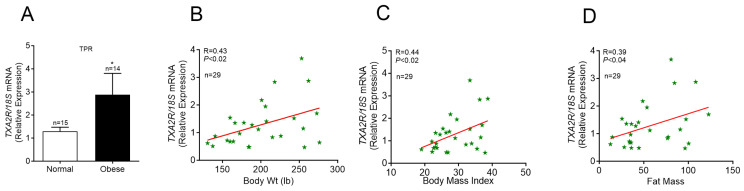
*TXA2R* expression levels in PBMCs from normal and obese subjects: (**A**) *TXA2R* mRNA expression in human PBMCs. Values are mean ± SEM. * *p* < 0.05 vs. Normal. Correlative analysis performed on normal and obese subjects (*n* = 29) comparing *TXA2R* gene expression with (**B**) body weight, (**C**) body mass index, and (**D**) fat mass.

We analyzed the mRNA levels of *TXA2R* (encoding TPR) in PBMCs from normal subjects and individuals with obesity (Figure 1A). The *TXA2R* mRNA level was significantly higher in PBMCs collected from subjects with obesity and positively correlated with body weight and fat mass (Figure 1B–D). These data suggest that a positive relationship exists between PBMC-TPR and obesity.

### 3.2. Blockade of TPR Signaling Reduces Lipopolysaccharide (LPS)-Induced Pro-Inflammatory Response in Human PBMCs 

**Figure 2:**
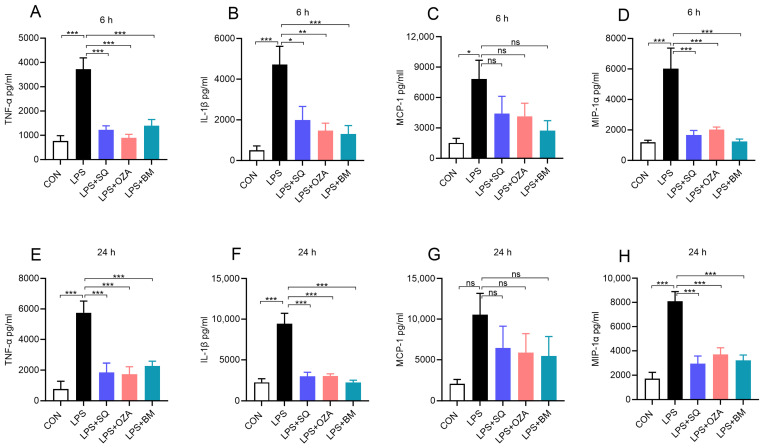
Inhibitors of TPR signaling reduced LPS-induced secretion of inflammatory mediators by human PBMCs. PBMCs were pre-treated with different agents inhibiting TPR signaling (10 µM) for 2 h, followed by co-incubation with LPS (10 ng/mL) for 6 h and 24 h. (**A**–**D**) Bar graphs show the amount of TNFα, IL-1β, MCP-1, and MIP-1α in cell culture supernatants at 6 h. (**E**–**H**) The levels of TNFα, IL-1β, MCP-1, and MIP-1α in cell culture supernatants after 24 h of treatment. Values are expressed as the mean ± SEM of 5 sets of experiments in duplicate (*n* = 10). * *p* < 0.05, ** *p* < 0.01, *** *p* < 0.001, and ns—not significant.

As mentioned, obesity is associated with an increase in circulating endotoxin levels [15]. Therefore, we sought to determine the impact of inhibiting TPR signaling on altering the LPS-induced pro-inflammatory response in PBMCs. As shown in Figure 2, our data show that LPS significantly increased the levels of TNFα, MCP-1, IL1β, and MIP-1α at 6 h (Figure 2A–D) and 24 h (Figure 2E–H). On the other hand, pretreatment with agents inhibiting TXA2 signaling, including SQ29548 (a TPR antagonist), Ozagrel (a TXA2 synthase inhibitor), and BM567 (a dual-acting agent inhibiting TXA2 synthase and TPR), attenuated the secretion of TNFα, IL1β, and MIP-1α. MCP-1 levels showed only a trend toward a decrease but not a significant decrease upon treatment with these agents (Figure 2A–H). We also measured the levels of IL6 and IL8, and we did not notice a significant difference among different groups. Next, we measured the levels of LDH, a marker of cytotoxicity, and we noted that the LDH levels did not alter among different groups, indicating that these agents did not induce cytotoxicity (Supplementary Figure S1). These findings indicate that TPR inhibitors effectively decrease the LPS-induced pro-inflammatory response in PBMCs. 

### 3.3. Inhibition of TPR Attenuates LPS-Induced TXB2 Secretion

Next, we wanted to check if LPS actually increases the formation of TXB2 in PBMCs. We analyzed the media supernatants for TXB2 levels and noted an increase in TXB2 release upon treatment with LPS. Interestingly, pre-treatment with a TPR antagonist and TXAS inhibitor, the dual-acting agent inhibiting both TPR and TXAS, inhibited LPS-induced TXB2 secretion into the media (Supplementary Figure S2). 

### 3.4. Blockade of TPR Signaling Reduces Stearic Acid-Induced Pro-Inflammatory Response in Human PBMCs 

**Figure 3:**
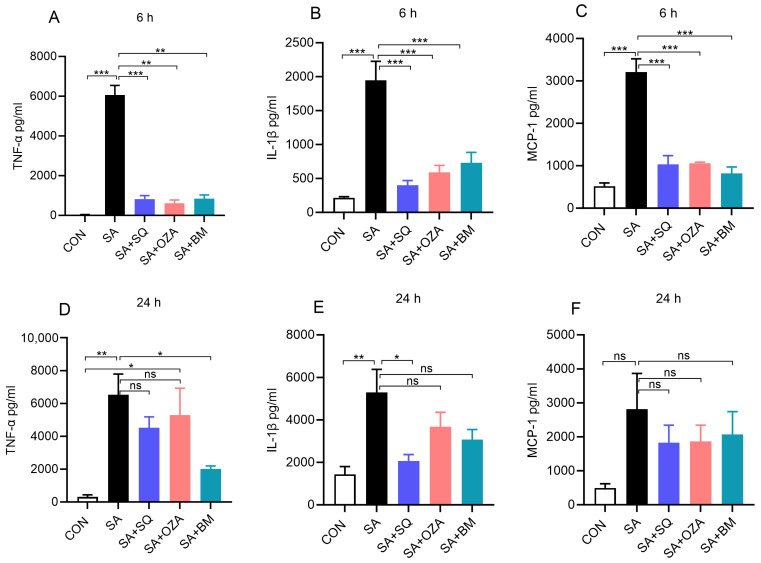
Inhibitors of TPR signaling reduced stearic acid (SA)-induced secretion of inflammatory mediators by human PBMCs. PBMCs were pre-treated with different agents inhibiting TPR signaling (10 µM) for 2 h, followed by co-incubation with SA (90 µM) for 6 h or 24 h. (**A**–**C**) Bar graphs show the amount of TNFα, IL-1β, and MCP-1 in cell culture supernatants at 6 h. (**D**–**F**) The levels of TNF-α, IL-1β, and MCP-1 in cell culture supernatants after 24 h of treatment. Values are expressed as the mean ± SEM of 3 sets of experiments in duplicate (*n* = 6). * *p* < 0.05, ** *p* < 0.01, *** *p* < 0.001, and ns—not significant.

Circulating plasma FFAs are increased in obesity and obesity-related comorbidities [18], and saturated fatty acids are known to exert an inflammatory response in obesity [19]. Therefore, we next studied the effects of TPR antagonism on SA-induced inflammatory responses in PBMCs. Our data clearly show that SA by itself led to a significant increase in the level of TNFα, IL1β, and MCP-1 at both 6 h (Figure 3A–C) and 24 h (Figure 3D–F) time points. BM567, the dual-acting agent, and SQ, a TPR antagonist, significantly reduced the SA-induced increase in TNFα, IL1β, and MCP-1, respectively. Inhibiting THAS alone using OZA showed a trend toward a decrease in these inflammatory mediators. Overall, these data suggest that the SA-induced inflammatory response can be attenuated by blocking TPR signaling. Further, we measured the levels of LDH, a marker of cytotoxicity, and noted that the LDH level did not change in any of the groups at 6 h. Although SA treatment led to an increase in LDH at 24 h, the inhibitors did not alter the LDH levels, indicating that the inhibitors block the SA-induced inflammatory response without altering cell viability (Supplementary Figure S3).

### 3.5. Activation of TPR Potentiates LPS- and SA-Induced Pro-Inflammatory Response in PBMCs

**Figure 4:**
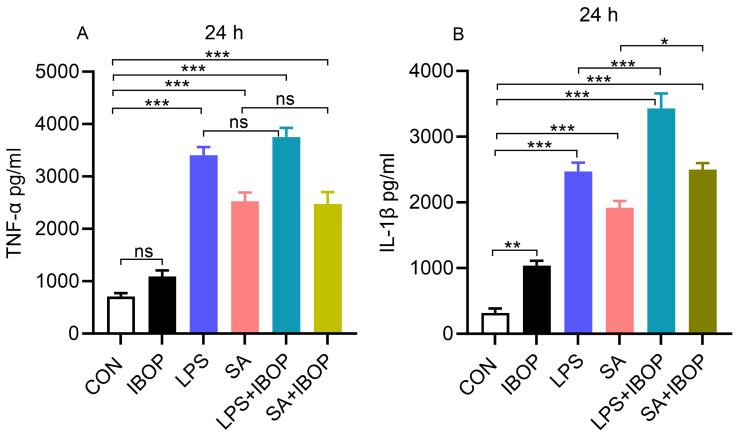
Thromboxane A2 receptor activation aggravates LPS- and SA-induced secretion of pro-inflammatory mediators by human PBMCs. PBMCs were pre-treated with IBOP (2 µM), a TPR agonist, for 2 h, followed by co-incubation with LPS or SA for 24 h. (**A**) Bar graphs show the level of TNFα in cell culture supernatants at 24 h. (**B**) The level of IL-1β in cell culture supernatants after 24 h of treatment. Values are expressed as the mean ± SEM of 3 sets of experiments in duplicate (*n* = 6). * *p* < 0.05, ** *p* < 0.01, *** *p* < 0.001, and ns—not significant.

PBMCs were pre-treated with IBOP, a TPR agonist, for 2 h and then stimulated with LPS or SA. We noticed that LPS or SA alone significantly increased the level of TNFα and IL1β in cultured PBMCs (Figure 4A,B). Interestingly, IBOP enhanced the pro-inflammatory effect of LPS and SA in PBMCs, as evident from the greater increase in IL1β (Figure 4B), further providing evidence that TPR activation promotes an inflammatory response in PBMCs.

### 3.6. BM567, a Dual-Inhibitor, Attenuates TPR Agonist-Mediated Inflammatory Response in PBMCs

**Figure 5:**
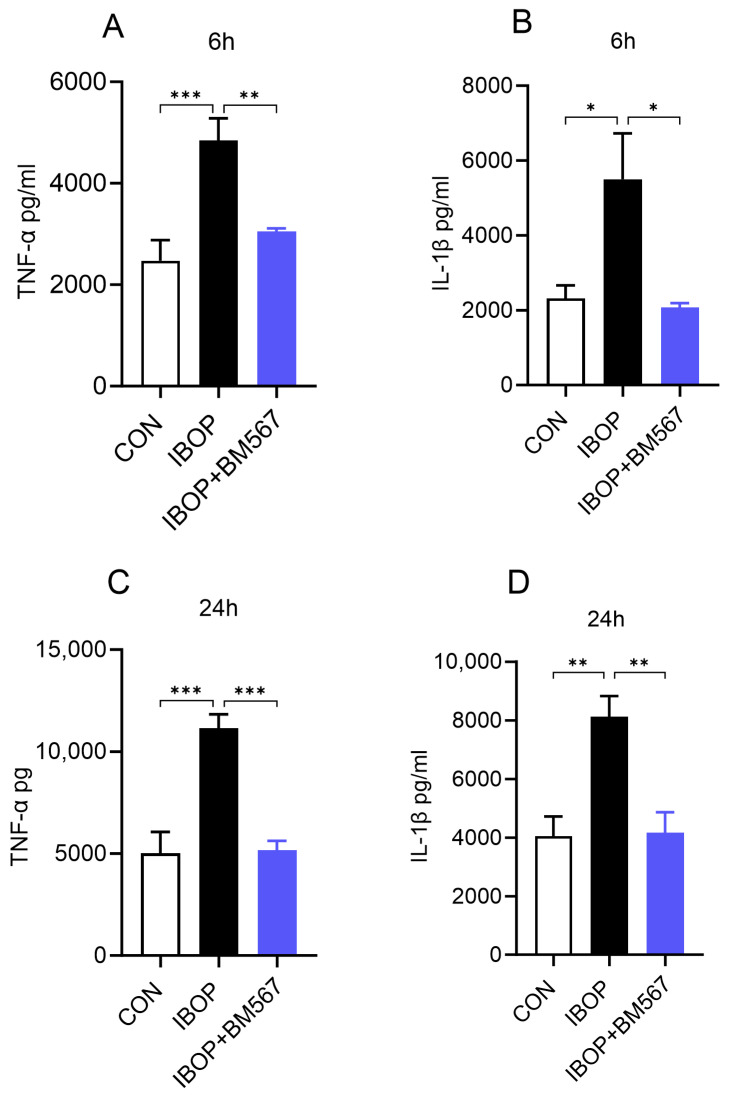
A dual-acting agent inhibiting TPR and TXA2 synthase inhibits IBOP-induced secretion of pro-inflammatory mediators by human PBMCs. PBMCs were pre-treated with BM567 (10 µM) for 2 h, followed by co-incubation with IBOP (2 µM) for 6 h and 24 h. (**A**,**B**) Bar graphs show the level of TNFα and IL-1β in cell culture supernatants at 6 h. (**C**,**D**) The level of TNFα and IL-1β in cell culture supernatants after 24 h of treatment. Values are expressed as the mean ± SEM of 3 sets of experiments in duplicate (*n* = 6). * *p* < 0.05, ** *p* < 0.01, and *** *p* < 0.001.

Next, we stimulated PBMCs with IBOP alone in the presence or absence of BM567, a dual-acting agent, to determine the specific effect of TPR activation on the PBMC inflammatory response. We noted that IBOP by itself significantly increased the levels of TNFα and IL1β in the media, and this effect was attenuated by BM567 (Figure 5A–D). These data show that the direct activation of TPR using its agonist can induce an inflammatory response, which can be blocked by inhibiting TPR signaling. 

### 3.7. Inhibition of ROCK Activity Blocks IBOP-Induced Pro-Inflammatory Response in PBMCs

**Figure 6:**
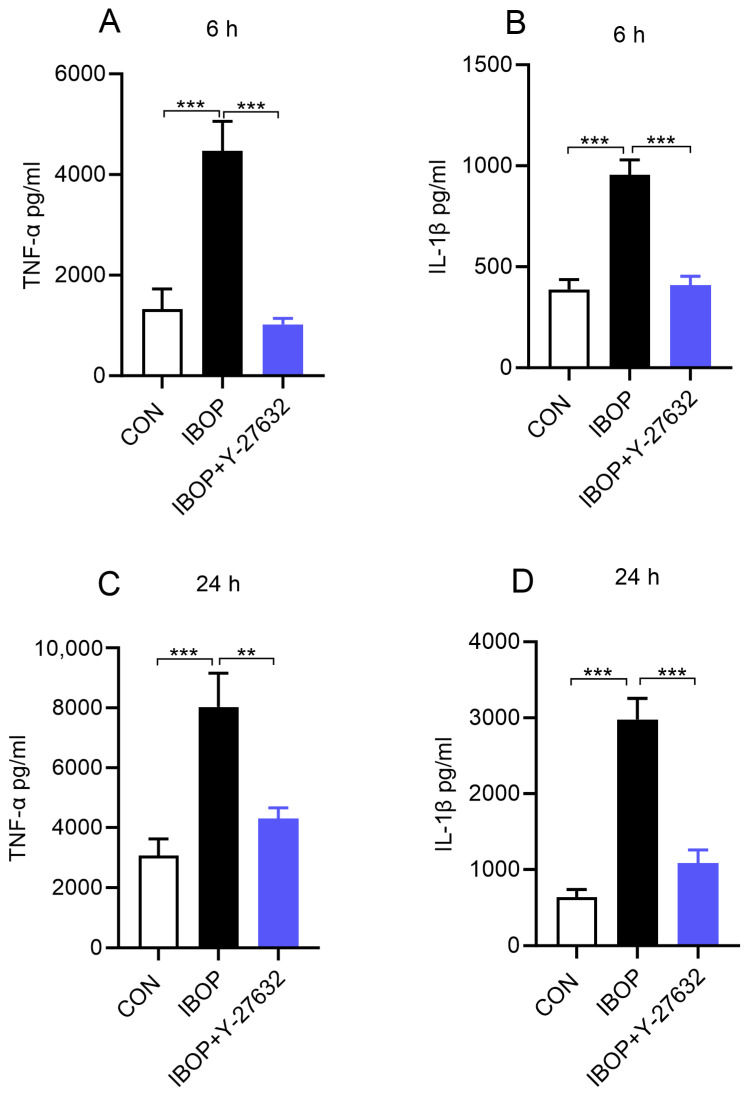
ROCK inhibition attenuates IBOP-induced secretion of pro-inflammatory mediators by human PBMCs. PBMCs were pre-treated with Y-27632, a ROCK inhibitor, for 2 h, followed by co-incubation with IBOP (2 µM) for 6 h and 24 h. (**A**,**B**) Bar graphs show the level of TNFα and IL-1β in cell culture supernatants at 6 h. (**C**,**D**) The level of TNFα and IL-1β in cell culture supernatants after 24 h of treatment. Values are expressed as the mean ± SEM of 4 sets of experiments (2–3 samples per group) (*n* = 10). ** *p* < 0.01, and *** *p* < 0.001, con vs. IBOP and IBOP vs. treatment groups.

Regarding potential mechanisms, a link exists between ROCK and TPR signaling, and ROCK1 is involved in promoting an inflammatory response. Therefore, we next treated PBMCs with IBOP, a TPR agonist, in the presence or absence of Y27632, a ROCK inhibitor. As shown in Figure 6, IBOP alone increased the release of TNFα and IL1β, and this effect is blocked by the ROCK inhibitor (Figure 6A–D). These data suggest that TPR promotes an inflammatory response via ROCK. 

## 4. Discussion

In the present study, we have demonstrated that a positive association exists between PBMC TPR and human obesity. Ex vivo studies in human PBMCs showed that LPS, a TLR4 agonist, and SA, an FFA, exerted a pro-inflammatory effect in PBMCs. Both LPS- and FFA-induced inflammatory responses are attenuated by blocking TPR signaling. Our studies show that IBOP, a TPR agonist, enhanced the LPS- or FFA-induced inflammatory response in PBMCs. Moreover, IBOP by itself induces a pro-inflammatory effect in PBMCs, which was attenuated by inhibiting TPR signaling. Interestingly, LPS-, FFA-, and IBOP-induced pro-inflammatory effects were also attenuated by inhibiting ROCK activity. Together, these data suggest that a link exists between PBMC TPR and human obesity and that TPR exerts pro-inflammatory effects in PBMCs via TLR4 and/or ROCK signaling. 

Although hemostasis and vasoconstriction are important processes regulated by TPR, several lines of evidence suggest that it plays a role in promoting inflammation in endothelial cells and macrophages. Several lines of evidence support the concept that obesity-induced inflammation plays an important role in the development of insulin resistance [20,21,22]. Macrophages are the predominant immune cell population in human AT. The percentage of macrophages in human AT is estimated to range from 10% in lean subjects to 40% in individuals with obesity [23]. A strong link exists between obesity and inflammation [3,24,25]. Of note, an elevated TXA2 to PGI2 (an anti-inflammatory lipid mediator) ratio in urine and blood plasma has been reported in human subjects and animals exhibiting metabolic syndrome [26,27]. However, evidence for the link between PBMC TPR signaling and obesity in humans is still lacking. We provide evidence that TPR expression is increased in the PBMCs collected from individuals with obesity compared to normal subjects. In addition, PBMC TPR expression is positively correlated to body weight, body mass index, and fat mass. 

The direct effects of altering TPR signaling on the inflammatory response in human PBMCs remain unknown. A great deal of emphasis has been placed on elucidating the pro-inflammatory effect of TPR in endothelial cells [5,6,7]. This is consistent with the well-known effects of TPR in mediating vasoconstriction. Although TPR is widely recognized as an inflammatory gene, limited studies are available on the role of TPR in altering macrophage inflammatory responses. Altavilla et al. reported that G619, a dual TXAS inhibitor and TPR antagonist, inhibits LPS-induced TNFα release in rats in vivo as well as isolated rat peritoneal macrophages [12]. In addition, TPR has been shown to promote a pro-inflammatory effect in microglia, which are resident macrophages in the brain, upon LPS treatment via the activation of MAPK and/or NFκB [13,14]. Our data are in line with these studies and provide evidence that, in addition to LPS, the SA-induced pro-inflammatory response is attenuated by blocking TPR. These data suggest that TPR plays an important role in mediating the pro-inflammatory effects of obesity-related factors, in particular LPS and SA, in human PBMCs. 

Regarding potential mechanisms, the fact that LPS is a TLR4 agonist and TPR antagonism attenuates the LPS-induced inflammatory response suggests that there may be coordination between TLR4 and TPR signaling in mediating a macrophage inflammatory response. In fact, the crosstalk between TPR and TLR4 signaling in canine platelets has been reported, where inhibition of TLR4 signaling attenuated LPS-induced TXA2 production and platelet activation [28]. Our data show that the pro-inflammatory effects invoked by LPS, a well-known TLR4 agonist, are attenuated by various agents blocking TPR signaling, including the TPR antagonist, THAS inhibitor, and a dual-acting agent blocking both the enzyme and the receptor, indicating the interaction between TLR4 and TPR in mediating an inflammatory response in PBMCs. 

It should also be noted that LPS- and SA-induced pro-inflammatory effects are enhanced by the TPR agonist, further supporting the notion that coordination exists between TLR4 and TPR signaling in mediating the inflammatory response. However, the involvement of other mechanisms in mediating the pro-inflammatory effects of TPR cannot be ruled out. Evidence suggests that ROCK is a key mediator of TPR, leading to endothelial cell dysfunction [29,30,31]. ROCK plays a role in regulating the migratory properties of bone-marrow-derived macrophages [32]. However, the role of ROCK in altering the PBMC inflammatory response remains unclear. Our data provide compelling evidence that inhibition of ROCK signaling using Y-27632 attenuates the LPS- and SA-induced inflammatory response. Moreover, Y-27932 inhibits the IBOP-induced inflammatory response, providing direct evidence for the role of ROCK in mediating the pro-inflammatory effect elicited by TPR activation. 

Our study has some limitations. For example, PBMCs are a mixed population of cells that contain T lymphocytes, B lymphocytes, and monocytes. T lymphocytes are the most predominant cells in human PBMCs. About 49–77% of PBMCs are T lymphocytes. B cells and monocytes account for 6–17% and 6–12%, respectively [33]. Studies have shown that TP-R is expressed on lymphocytes, and stimulation of TP-R promotes lymphocyte activation [10,34]. It is likely that T lymphocytes may be the main source of TP-R in PBMCs, as they are the most abundant cells in a PBMC population. However, the role of TP-R in mediating a pro-inflammatory effect in B lymphocytes and monocytes cannot be ruled out. Although the contribution of individual cells in mediating the pro-inflammatory effects is unclear, PBMCs are the most commonly used immune cells, which serve as a relevant model to study the mechanisms involved in the inflammatory response in humans. Further studies are warranted to determine the role of TPR, specifically in lymphocytes or monocytes, in mediating the inflammatory response. Next, PBMCs from normal subjects and individuals with obesity and/or insulin resistance were used in ex vivo experiments. The inter-individual variability and genetic influence on cytokine responses of PBMCs to inflammatory stimuli are possible [35]. However, our data from individual experiments are derived from PBMCs collected from at least three subjects. 

Taken together, our data suggest that an association exists between PBMC TPR and human obesity, and blocking TPR signaling attenuates the LPS- and/or FFA-induced inflammatory response in PBMCs. Further, our data indicate that TLR4 and ROCK1 signaling play a role in mediating the effects of TPR on inflammatory processes. Blocking TPR activity may be a therapeutic option to attenuate obesity-related inflammation and metabolic disorders. TP-R antagonists are being used to manage asthma, arterial thrombosis, and peripheral artery disease in some Asian and European countries [36,37]. Several TP-R antagonists, including Ifetroban, are being studied in various phases of preclinical and clinical investigations [36]. Our findings will be relevant to testing the effectiveness of TP-R antagonists against obesity-related comorbidities. 

## Figures and Tables

**Table 1:** Baseline characteristics.

Measurement	Normal (*n* = 15)	Obesity (*n* = 14)	*p* Value
Age (years)	40.20 ± 4.27	47.43 ± 2.22	0.1537
Weight (lbs)	168.7 ± 5.80	241.1 ± 7.15	<0.0001
Body mass index (kg/m^2^)	24.24 ± 0.61	34.79 ± 0.96	<0.0001
Insulin (µIU/mL)	4.53 ± 1.02	12.85 ± 3.64	0.0275
Glucose (mg/dL)	90.00 ± 1.60	92.00 ± 2.67	0.5206
HOMA-IR	1.01 ± 0.23	2.88 ± 0.71	0.0144
Total cholesterol (mg/dL)	197.4 ± 6.66	208.3 ± 14.54	0.4922
Triglycerides (mg/dL)	114.8 ± 18.23	168.8 ± 42.18	0.2394
LDL cholesterol (mg/dL)	121.3 ± 6.92	140.5 ± 13.21	0.1941
HDL cholesterol (mg/dL)	53.13 ± 3.65	41.07 ± 2.67	0.0140
Creatinine (mg/dL)	1.05 ± 0.03	1.02 ± 0.05	0.7158
ALT (U/L)	17.80 ± 2.88	38.07 ± 13.29	0.1353
AST (U/L)	31.00 ± 2.12	34.64 ± 4.51	0.4616
HA1C (%)	5.43 ± 0.51	5.77 ± 1.85	0.0063
GFR (mL/min)	82.87 ± 4.08	82.36 ± 5.03	0.9375
CRP (mg/L)	2.69 ± 1.03	2.94 ± 0.68	0.8442
Fat mass (%)	19.53 ± 1.14	35.51 ± 1.32	<0.0001

Values are mean ± SEM. HOMA-IR, Homeostatic Model Assessment for Insulin Resistance; ALT, alkaline phosphatase; AST, acid phosphatase.

## Data Availability

The raw data supporting the conclusions of this article will be made available by the authors upon request.

## Supplementary Materials

The following supporting information can be downloaded at https://www.mdpi.com/article/10.3390/cells13161320/s1, Supplementary Figure S1: LDH cytotoxicity assay; Supplementary Figure S2: Thromboxane B2 (TXB2) assay; Supplementary Figure S3: LDH cytotoxicity assay.
