# Supplementary material for: Blocking Thromboxane-Prostanoid Receptor Signaling Attenuates Lipopolysaccharide- and Stearic Acid-Induced Inflammatory Response in Human PBMCs
Source: Cells. 2024 Aug 8;13(16):1320. doi: 10.3390/cells13161320 (PMC11352481; doi:10.3390/cells13161320)
Supplement: Supplementary file 1 [file cells-13-01320-s001.zip › cells-3116334-supplementary.pdf]

Blocking Thromboxane-Prostanoid Receptor Signaling Attenuates Lipopolysaccharide and Stearic Acid-Induced Inflammatory Response in Human PBMCs

Supplementary Figures

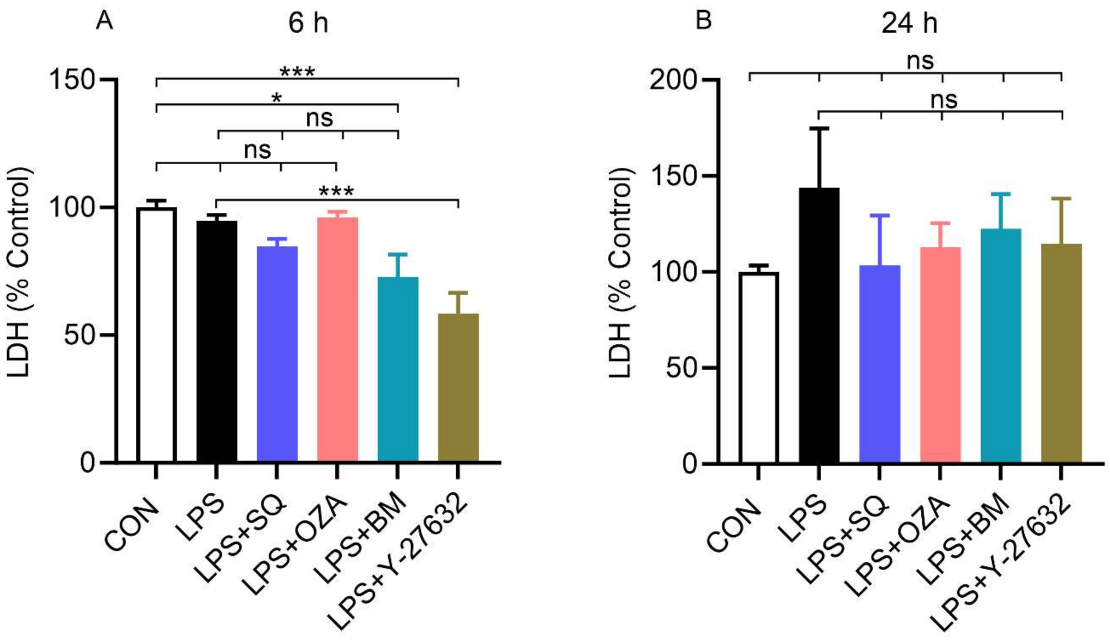

Supplementary Figure S1. LDH cytotoxicity assay: (A&B) Bar graphs show the level of LDH released into the media of PBMCs treated with LPS in the presence or absence of various inhibitors for 6 h and 24 h. Values are expressed as mean ± SEM of 3 sets of experiment in duplicate (n=6). *P<0.05, ***P<0.001 and ns-not significant.

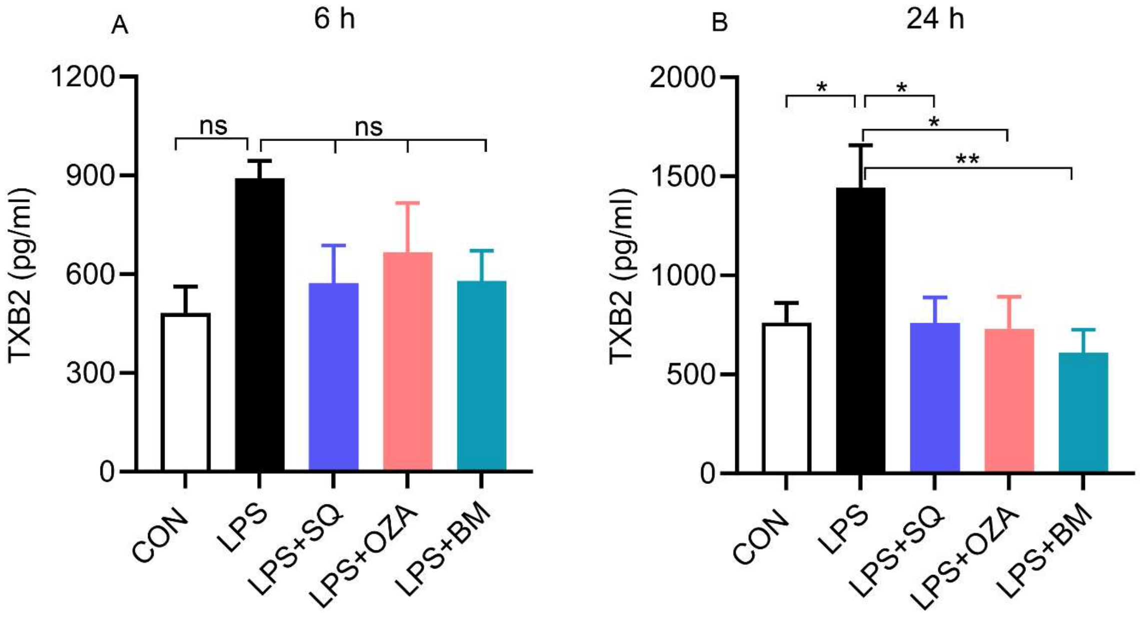

Supplementary Figure S2. Thromboxane B2 (TXB2) assay: Bar graphs show the levels of TXB2 released into the media of PBMCs treated with LPS in the presence or absence of various inhibitors for 6 h and 24 h. Values are expressed as mean ± SEM of 3 sets of experiment in duplicate (n=6). **P<0.01, ***P<0.001 and ns-not significant.

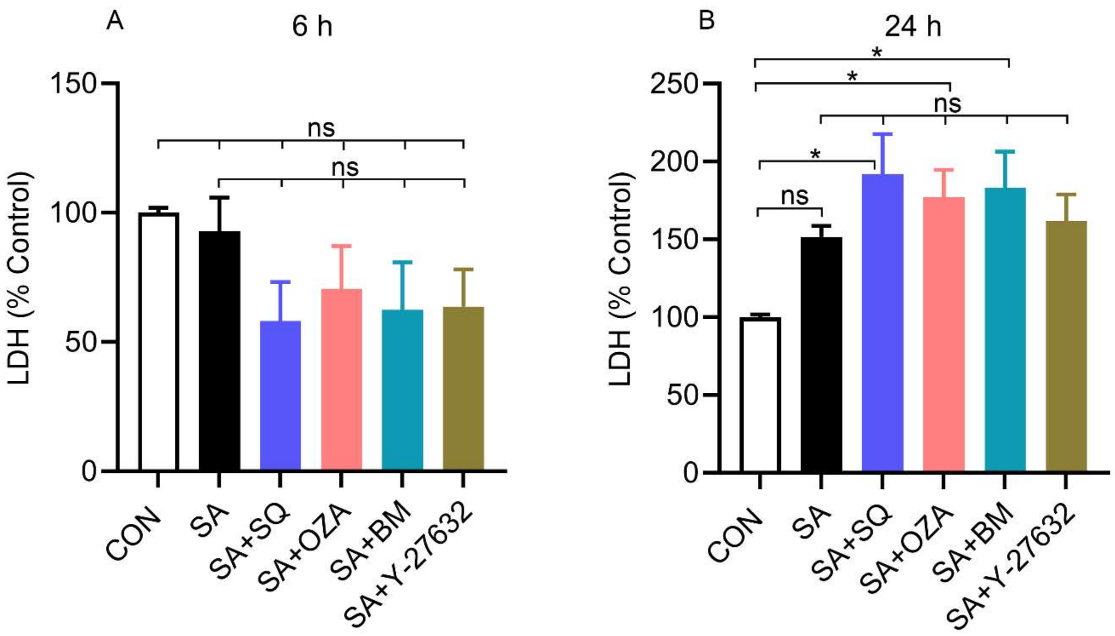

Supplementary Figure 3. LDH cytotoxicity assay: Bar graph shows the level of LDH released into the media of PBMCs treated with SA in the presence or absence of various inhibitors for 6 h and 24 h. Values are expressed as mean ± SEM of 3 sets of experiment in duplicate (n=6). *P<0.05 and ns-not significant.
